# A novel tumor-homing TRAIL variant eradicates tumor xenografts of refractory colorectal cancer cells in combination with tumor cell-targeted photodynamic therapy

**DOI:** 10.1080/10717544.2022.2079766

**Published:** 2022-05-30

**Authors:** Zhao Li, Tianshan She, Hao Yang, Tao Su, Qiuxiao Shi, Ze Tao, Yanru Feng, Fen Yang, Jingqiu Cheng, Xiaofeng Lu

**Affiliations:** a NHC Key Lab of Transplant Engineering and Immunology, West China Hospital, Sichuan University, Chengdu, China; b Sichuan Provincial Engineering Laboratory of Pathology in Clinical Application, West China Hospital, Sichuan University, Chengdu, China; c Frontiers Science Center for Disease-related Molecular Network, West China Hospital, Sichuan University, Chengdu, China; d Laboratory of Clinical Proteomics and Metabolomics, Institutes for Systems Genetics, West China Hospital, Sichuan University, Chengdu, China

**Keywords:** Tumor necrosis factor-related apoptosis-inducing ligand, photodynamic therapy, cancer-targeted therapy, multidrug resistance, colorectal cancer

## Abstract

Multidrug resistance (MDR), which is common in colorectal cancer (CRC), induces high mortality in patients. Due to its robust and selective apoptosis induction in some CRC cells with MDR, tumor necrosis factor-related apoptosis-inducing ligand (TRAIL) is attractive as a novel tool for CRC therapy. However, TRAIL is limited by its poor tumor-homing ability and inefficient apoptosis induction in CRC cells expressing low levels of death receptor (DR). Here, the tumor-homing RGR peptide (CRGRRST) was fused to TRAIL to produce RGR-TRAIL. Compared with TRAIL, RGR-TRAIL showed greater cell binding and cytotoxicity in CRC cells. In addition, RGR-TRAIL exerted significantly enhanced tumor uptake and growth suppression in mice bearing CRC tumor xenografts. Notably, RGR-TRAIL eradicated all tumor xenografts of DR-overexpressing COLO205 cells. However, TRAIL only showed mild tumor growth suppression under the same conditions, indicating that RGR fusion significantly increased the antitumor effect of TRAIL in DR-overexpressing CRC cells by improving tumor homing. Nevertheless, RGR fusion did not significantly enhance the antitumor effect of TRAIL in HT29 cells expressing low levels of DR. We found that DR expression in HT29 cells was enhanced by epidermal growth factor receptor (EGFR)-targeted photodynamic therapy (PDT). Moreover, both the *in vitro* and *in vivo* antitumor effects of RGR-TRAIL were significantly improved by combination with PDT. HT29 tumor xenografts (∼20%) were even eradicated by combination therapy. These results indicate that it is valuable to further evaluate the combination therapy of RGR-TRAIL and tumor-targeted PDT for clinical therapy of CRC with MDR.

## Introduction

Colorectal cancer (CRC) ranked as the third most common cancer and second in terms of mortality worldwide in 2020 (Sung et al., 2021). The five-year survival rates for CRC patients at stages I and II are above 60%. However, the survival rate drops to 14% in CRC patients at stage III or later (advanced stages) (Siegel et al., 2020). In addition, over 50% of CRC patients are diagnosed at an advanced stage (McQuade et al., 2017). It is well known that conventional chemotherapies for CRC are limited by their severe systemic toxicities (Gustavsson et al., 2015). An increasing number of molecular targeted therapies with reduced nonspecific toxicities have been developed in recent years (Xie et al., 2020). However, CRC cells easily develop multidrug resistance (MDR) to both conventional chemotherapies and molecular targeted therapies, owing to the high expression of ATP-binding cassette-(ABC) transporters in enterocytes (Micsik et al., 2015; Cao et al., 2017; Xue et al., 2019). Protein drugs such as monoclonal antibodies and antitumor proteins may bypass pathways for MDR. Nevertheless, heterogenic mutations in genes, such as PIGF, RAS/RAF, and PI3K/AKT, induce CRC resistance to antibodies against vascular endothelial growth factor receptor (VEGFR) and epithelial growth factor receptor (EGFR) (Xie et al., 2020), indicating the urgent need to develop innovative strategies to combat CRC with MDR precisely and effectively.

Tumor necrosis factor-related apoptosis-inducing ligand (TRAIL), an antitumor protein, can trigger death receptors, including DR5 and DR4, to induce apoptosis of cancer cells at low nanomolar concentrations. However, due to the high expression of decoy receptors (DcR1 and DcR2), normal cells are protected from TRAIL. The preferential cytotoxicity in cancer cells makes TRAIL an attractive anticancer agent candidate. However, although recombinant TRAIL has shown super cytotoxicity in many cancer cells *in vitro*, its antitumor effect is far from satisfactory in cancer patients as monotherapy (Fulda, 2015). To improve the *in vivo* antitumor effect of TRAIL by increasing its tumor accumulation, tumor-homing molecules, such as antibodies or peptides recognizing some tumor-associated antigens, were fused to TRAIL to produce tumor-homing TRAIL variants (Tao et al., 2017; Dianat-Moghadam et al., 2020; Snajdauf et al., 2021). In fact, these tumor-homing TRAIL variants exerted enhanced antitumor effects in preclinical trials, indicating that tumor-targeted delivery is an alternative way to improve the antitumor effect of TRAIL.

Nevertheless, many cancer cells, including some CRC cells, still show resistance to native TRAIL and tumor-homing TRAIL variants (Deng & Shah, 2020), suggesting the need for the combination of TRAIL with other drugs or therapies. Although synergy between chemical drugs and TRAIL in killing cancer cells has been extensively reported *in vitro* (Stolfi et al., 2012; Wong et al., 2019), combined with chemical drugs did not improve the antitumor effect of TRAIL in CRC patients, which was predominantly attributed to the limitation of chemical drugs in sensitizing cancer cells to TRAIL (Wainberg et al., 2013). In fact, due to nonspecific toxicity, only a low dose of chemical drugs could be used for combination therapy with TRAIL. It appeared to be better to combine TRAIL with tumor-targeted therapies that can sensitize tumor cells to TRAIL but spare normal cells. In fact, photodynamic therapy (PDT) mediated by the injection of a tumor-targeted photosensitizer (PS) followed by tumor-restricted illumination could precisely damage tumor tissues (Manghnani et al., 2018). Moreover, in our previous studies, PDT was observed to sensitize CRC cells to TRAIL by upregulating death receptors and/or downregulating anti-apoptotic proteins in tumor cells. Combination therapy of a long-acting TRAIL variant and tumor cell-targeted PDT exerted promising antitumor effects in mice bearing tumor grafts of different CRC cells (Moan & Berg, 1991; Dysart & Patterson, 2005; Mitsunaga et al., 2011; She et al., 2021). Nevertheless, the long-acting TRAIL variant showed poor tumor-homing ability, which is not ideal for clinical translation. Therefore, we hope to evaluate the combination therapy of a tumor-homing TRAIL variant and tumor cell-targeted PDT in combating CRC with both chemotherapeutic MDR and TRAIL resistance.

In this study, a tumor-homing TRAIL variant, RGR-TRAIL, was first produced by fuzing the tumor-homing RGR peptide (Johansson et al., 2012) to TRAIL. Subsequently, the antitumor effect of RGR-TRAIL was evaluated in mice bearing tumor xenografts of different CRC cells with chemotherapeutic MDR. In addition, tumor cell-targeted PDT was developed by injection of EGFR-targeted PS followed by tumor-restricted irradiation. Finally, the synergy between RGR-TRAIL and EGFR-targeted PDT was evaluated in mice bearing tumor grafts of CRC cells with both chemotherapeutic MDR and TRAIL resistance. Our results demonstrated that combination therapy of a tumor-homing TRAIL variant and tumor cell-targeted PDT might be developed as a novel strategy to combat refractory CRC.

## Materials and methods

### Bioinformatics analysis of MDR-associated genes in CRC tissues

To estimate the MDR of CRC, the expression of ABC transporters in CRC tissues derived from patients was determined by bioinformatics analysis. Briefly, RNA-seq data of colon (445) and rectal (165) cancer samples were downloaded from The Cancer Genome Atlas (TCGA) database. RNA-seq data for 779 normal tissues were obtained from the Genotype-Tissue Expression (GTEx) database. The expression levels of ABC transporters in CRC and normal tissues were analyzed and compared using Gene Expression Profiling Interactive Analysis (GEPIA, http://gepia.cancer-pku.cn/), a web-based tool to deliver fast and customizable functionalities based on TCGA and GTEx data (Tang et al., 2017). The expression level of genes in raw data from different sources was normalized according to a standard pipeline provided by the UCSC Xena project (http://xena.ucsc.edu/). Simultaneously, to evaluate the sensitivity of CRC cells to TRAIL, the expression of death receptors in these same CRC tissues was also analyzed and compared to that in normal tissues.

### Expression and purification of proteins

The recombinant TRAIL and the EGFR-specific affibody (Ze) were prepared according to our previous work (Li et al., 2016; She et al., 2021). To produce RGR-TRAIL, the tumor-homing peptide RGR (CRGRRST) was fused to the N-terminus of TRAIL with a flexible linker (G4S)_3_. To construct the expression plasmid, the gene encoding RGR-TRAIL was synthesized by GenScript (Nanjing, China) and cloned into pQE30. The 6His-tag in the original pQE30 plasmid was replaced by a (HE)_3_-tag. The RGR-TRAIL expression plasmid was transformed into *Escherichia coli* (*E. coli*) M15 and induced by the addition of isopropyl-L-thio-β-D-galactopyranoside (IPTG, 0.1 mM) at 25 °C overnight. Subsequently, the cells were collected by centrifugation at 7000 g and resuspended in lysis buffer (50 mM phosphate, pH 8.0, 300 mM NaCl, 10 mM β-mercaptoethanol, 5 mM imidazole and 1 mM phenylmethylsulfonylfluoride) followed by processing in a high-pressure homogenizer (70–80 MPa). The recombinant proteins were recovered from the supernatant of *E. coli* M15 using Ni-NTA affinity chromatography according to the protocol provided by the manufacturer (Qiagen, CA). The purity and molecular weight of recovered proteins were determined by sodium dodecyl sulfate–polyacrylamide gel electrophoresis (SDS–PAGE) with a gel of 12.75% (for TRAIL and RGR-TRAIL) or a gel of 16% (for Ze affibody). The purified proteins were dialyzed against phosphate-buffered saline (PBS, 10 mM Na_2_HPO_4_, 2 mM KH_2_PO_4_, 137 mM NaCl, 2.68 mM KCl, pH 7.4) at 4 °C overnight. The dialyzed proteins were stored at −70 °C for further use.

### Cell culture and cytotoxicity assays

Human cancer cells (COLO205, LS174T, HCT116, HT29, HCT8, MDA-MB-231, MCF-7, A549, NIC-H460, HuCCT1, DU145, HT1080, U251, A375) and human umbilical vein endothelial cells (HUVECs) were purchased from the American Type Culture Collection (ATCC, VA). Human colorectal cancer cells (LIM1215) were purchased from the European Collection of Authenticated Cell Cultures (ECACC, Salisbury, UK). These cells were cultured in DMEM or RPMI 1640 medium supplemented with 10% fetal bovine serum (FBS), 2 mM L-glutamine, 100 U/mL penicillin, and 100 μg/mL streptomycin at 37 °C in a 5% CO_2_ humidified atmosphere.

To measure the cytotoxicity of chemical drugs (cisplatin, vincristine, doxorubicin, and bortezomib) (Selleck, TX) and TRAIL proteins, 1–2 × 10^4^ cells in a 100 μL medium were seeded in the wells of 96-well plates and incubated overnight. Subsequently, TRAIL proteins or chemical drugs at different concentrations were added to these cells. After treatment overnight, the viable cells were measured using a Cell Counting Kit-8 (CCK8, Dojindo, Japan). PBS was used as a negative control, and the viability of PBS-treated cells was considered 100%. The 50% inhibitory concentrations (IC50s) of TRAIL proteins or chemical drugs were calculated according to their perspective curves for cell viability.

### Apoptosis assays

Annexin V and propidium iodide (PI) dual staining combined with flow cytometry was used to indicate apoptotic cells induced by TRAIL proteins *in vitro* according to our previous works (She et al., 2021). Briefly, 3 × 10^5^ cells in 100 μL media were treated with TRAIL proteins (0, 0.5, 10 nM) at 37 °C for 1 h. Subsequently, the cells were washed twice with PBS followed by dual staining with FITC-Annexin V and PI according to the manual provided by the manufacturer. Annexin V^+^/PI^-^ and Annexin V^+^/PI^+^ cells indicated by flow cytometry were considered early and late apoptotic cells, respectively. To further verify the involvement of caspases in apoptosis, the activity of caspase in cells treated with or without TRAIL proteins was measured by specific substrates (Promega, WI) for caspase 3, 8, and 9 according to the manual provided by the manufacturer. In addition, the activation of caspase 3, caspase 8, caspase 9, and PARP triggered by TRAIL proteins was also determined by western blot analysis of cytoplasmic proteins from cancer cells treated with or without TRAIL proteins. Western blotting was performed with specific antibodies against the cleaved forms of caspases and PARP and their corresponding secondary antibodies. β-actin was used as a loading control. The primary antibodies used included rabbit anti-human caspase 3 (ZEN BIO, Chengdu, China), mouse anti-human caspase 8 (Cell Signaling Technology, MA), rabbit anti-human caspase 9 (Cell Signaling Technology, MA), rabbit anti-human PARP (ZEN BIO, Chengdu, China), and mouse anti-human β-actin (Biolegend, CA). The protein bands were visualized using HRP-labeled corresponding secondary antibodies with TMB as substrate.

Caspase inhibitors were also used to determine the involvement of caspases in TRAIL protein-induced apoptosis according to our previous works (Tao et al., 2017). Briefly, in the cytotoxicity assay system, cells were incubated with or without caspase inhibitors at 37 °C for 2 h prior to the addition of TRAIL proteins into these cells. Subsequently, the viabilities of cells treated with TRAIL proteins in the presence of caspase inhibitors (20 μM)) were measured and compared to those of cells treated with TRAIL proteins in the absence of caspase inhibitors. The caspase inhibitor-induced increase in cell viability reflects the involvement of caspases in TRAIL protein-induced apoptosis. Both pan-caspase inhibitors and caspase 3-, 8-, and 9-specific inhibitors (Promega, WI) were used in this experiment. To examine apoptotic cells in tumor xenografts, mice bearing tumor xenografts were treated with monotherapy or combination therapy of TRAIL proteins and PDT on the first day. On the second day, tumor xenografts were collected, and apoptotic cells were indicated by terminal deoxynucleotidyl transferase dUTP nick end labeling (TUNEL, Promega, WI) according to the manual provided by the manufacturer.

### Death receptor binding assays

Biolayer interferometry performed on Octet systems (Pall Forte Bio LLC, CA) was used to measure the affinity of TRAIL proteins for death receptors. The death receptor proteins DR4-Fc and DR5-Fc (Sino Biological, Beijing, China) were immobilized on a protein A-coated biosensor followed by insertion into solutions containing different concentrations of TRAIL proteins for association according to our previous works (Yang et al., 2018). In addition, death receptor binding was also measured by neutralizing the cytotoxicity of TRAIL proteins. Briefly, in the cytotoxicity assay system, TRAIL proteins (2.5–50 nM) were mixed with DR4-Fc or DR5-Fc at different molar ratios (DR protein: TRAIL = 0–3) prior to addition to cells. The viabilities of cells treated with TRAIL proteins in the presence or absence of death receptor proteins were measured and compared. The increase in the viability of cancer cells after treatment with TRAIL proteins in the presence of death receptor proteins reflects the binding of TRAIL proteins to death receptors.

### Receptor expression and protein cell-binding assays

To detect the expression of DR4, DR5 and EGFR in cultured cancer cells, approximately 3 × 10^5^ cells in 100 μL PBS were incubated with primary antibodies at 4 °C for 1.5 h. After three washes with PBS containing 0.5% FBS, these cells were further incubated with corresponding secondary antibodies for an additional 0.5 h followed by analysis on a Cytomics FC500 (Backman, CA).

To evaluate the impact of PDT on the expression of DR4 and DR5 in cultured cancer cells, membrane proteins of cancer cells treated with or without PDT were extracted using RIPA lysis buffer (Beyotime, Shanghai, China) for SDS–PAGE followed by western blotting with rabbit anti-human DR4 or DR5 (Abcam, MN). PDT-mediated cytotoxicity is predominantly attributed to reactive oxygen species (ROS) (Alzeibak et al., 2021). To verify the involvement of ROS in PDT-mediated regulation of death receptors, the antioxidant acetylcysteine (NAC) (4 mM, Selleck Chemicals, TX) was added to the cells 1 h before PDT. To determine the impact of PDT on the expression of death receptors in tumor tissues, tumor xenografts treated with or without PDT were collected for immunofluorescence histochemistry with antibodies against DR4 and DR5. The primary antibodies used included rabbit anti-human EGFR, DR4, or DR5 (Abcam, MN), and the secondary antibodies labeled with DyLight 550 or DyLight 488 were derived from donkeys or goats (Abcam, MN).

To measure the cell binding of proteins to cultured cells, TRAIL proteins and Ze affibody were labeled with FAM or IR700 according to our previous works (She et al., 2021) prior to incubation with cells at 4 °C or 37 °C for 1 h. Subsequently, the bound proteins were measured by flow cytometry.

### Tumor uptake and tissue distribution of proteins

To monitor the tumor uptake of proteins by optical imaging, TRAIL, RGR-TRAIL, and Ze affibody were labeled with CF^TM^ 750 succinimidyl ester according to our previous work (Tao et al., 2017). Subsequently, CF750-labeled proteins were intravenously injected into mice bearing tumor xenografts followed by dynamic scanning using an IVIS optical imaging system (Caliper Life Science, CA). To evaluate the impact of PDT on tumor uptake of RGR-TRAIL, CF750-labeled RGR-TRAIL (5 mg/kg) was intravenously injected into mice bearing tumor xenografts immediately following by PDT. After the last scanning, the mice were sacrificed, and the tumor xenografts and normal organs/tissues were removed and scanned. To verify the protein-dependent accumulation, CF750-labeled Ze affibody (3 mg/kg) was digested by trypsin prior to injection into the mice.

To localize the proteins in tumor tissues, FAM-labeled proteins were intravenously injected into mice bearing tumor xenografts. The tumor xenografts were collected and sectioned under frozen conditions within 4 h postinjection. Tumor cells and endothelial cells were indicated by antibodies against EGFR and CD31 with their corresponding secondary antibodies (Biolegend, CA), respectively. Tumor tissues were incubated with the primary antibody at 37 °C for 1.5 h followed by three washes prior to incubation with the corresponding secondary antibody at 37 °C for an additional 0.5 h. The nuclei of the cells were visualized by DAPI prior to observation under a multiphoton laser confocal microscope (Nikon, Tokyo, Japan).

### Photodynamic therapy

To prepare the EGFR-targeted photosensitizer Ze-IR700, IRDye® 700DX N-hydroxysuccinimide ester (IR700, LI-COR Biosciences, Lincoln, NE) was conjugated to the Ze affibody according to our previous work (She et al., 2021). To perform the *in vitro* PDT, 2 × 10^4^ CRC cells were incubated with 1 μM Ze-IR700 in 96-well plates at 37 °C for 0.5 h. After two washes with RPMI 1640 medium, the cells were irradiated at a fluence rate of 16 mW/cm^2^ for a total dose of 10 J/cm^2^ with a laser at a wavelength of 690 nm. Ze-IR700 digested with trypsin (Ze-IR700 + TN) was used as a control. The surviving cells were measured on the next day using CCK-8. In addition, dual staining with SYTO 9 and PI (Invitrogen, CA) was also used to visualize live and dead cells. ROS produced by Ze-IR700-mediated PDT were detected by dichlorodihydrofluorescein diacetate (DCFH, Beyotime, Shanghai, China) and singlet oxygen sensor green (SOSG, Invitrogen, Carlsbad, CA) according to Shi et al. (2017). To investigate the synergy between TRAIL proteins and PDT, the cytotoxicity of TRAIL proteins in CRC cells pretreated with or without PDT was measured and compared. For *in vivo* PDT, 1 mg/kg Ze-IR700 was intravenously injected into mice followed by irradiation at a fluence rate of 100 mW/cm^2^ for a total dose of 60 J/cm^2^ at 4 h postinjection.

### Animal models and treatments

To produce animal models bearing tumor xenografts, CRC cells (2 × 10^6^ cells for COLO205, HCT116, and LS174T, 5 × 10^6^ cells for HT29) were subcutaneously injected into Balb/c nude mice. To monitor the growth of tumor grafts by optical imaging in some experiments, CRC cells overexpressing red fluorescent proteins (RFP, Ji Manchu Biotechnology, Shanghai, China) were subcutaneously injected into mice. Once the tumor xenografts were palpable, their longitudinal (L) and transverse (W) diameters were measured to calculate the tumor volume (V) according to the following formula: *V* = L × W × W/2. When the average tumor volume reached 100–150 mm^3^, the mice were randomly divided into several groups and treated with monotherapy or combination therapy of TRAIL proteins (5 mg/kg) and Ze-IR700-mediated PDT. For the combination therapy, TRAIL proteins were intravenously injected into mice prior to irradiation. The mice in the control group were treated with PBS. After treatment, the tumor volume and body weight of the mice were measured daily. The tissue damage in the tumor graft, kidney and liver was evaluated by H&E staining.

### Statistical analysis

One-way analysis of variance (ANOVA) for multiple comparisons was performed using SPSS software version 18.0. The results are expressed as the mean ± standard error (SD), and the significance level was defined as *P* < .05 (*), *P* < .01 (**), and *P* < .001 (***).

## Results

### TRAIL exerts cytotoxicity in CRC cells with chemotherapeutic multidrug resistance

**Figure 1.**
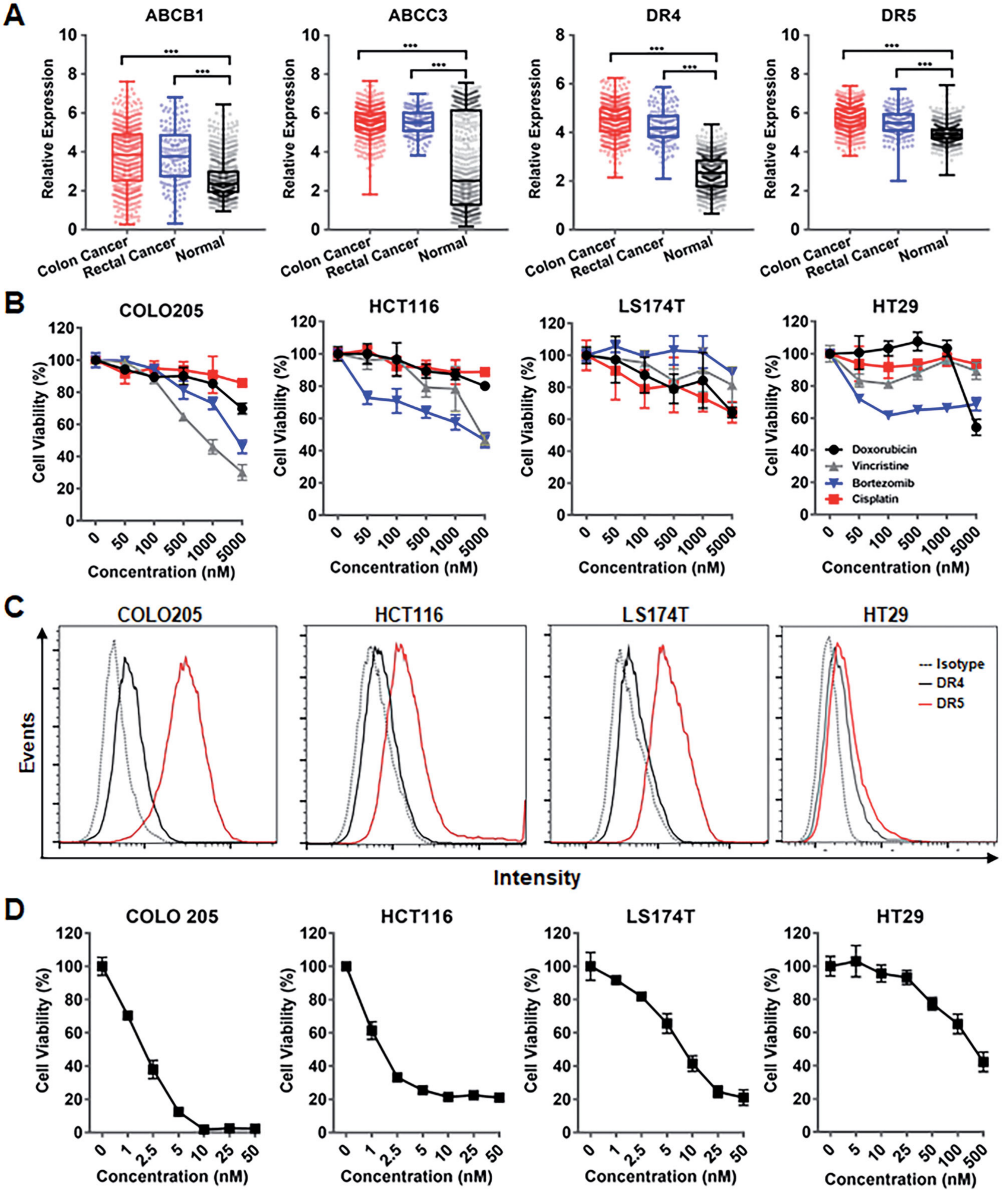
TRAIL exerted cytotoxicity in CRC cells with chemotherapeutic MDR. (A) Expression of ABC transporters contributing to chemotherapeutic MDR and the death receptors DR4 and DR5 for TRAIL in tumor tissues derived from CRC patients. (B) Cytotoxicity of chemical drugs in CRC cells. (C) Expression of the death receptors DR4 and DR5 in CRC cells. (D) Cytotoxicity of TRAIL in CRC cells with chemotherapeutic MDR.

The chemotherapeutic MDR of cancer cells is predominantly attributed to overexpressed chemical efflux pumps, such as ABC transporters. To investigate the expression of ABC transporters in tumor tissues derived from CRC patients, public RNA-seq data obtained from TCGA and GTEx were analyzed by GEPIA. As shown in Figure 1(A) and Supplementary Figure S1, bioinformatics analysis demonstrated that the tumor tissue levels of ABC transporters, including ABCB1, ABCB7, ABCC1, ABCC2, ABCC3, ABCC4, ABCC6, ABCC10, ABCC11, and ABCG1, were significantly higher than those in normal tissues, suggesting the MDR of CRC cells. In fact, cytotoxicity assays revealed that CRC cells, such as COLO205, HCT116, LS174T, and HT29, showed resistance (IC50 > 1000 nM) to all tested chemical drugs, including cisplatin, vincristine, doxorubicin, and bortezomib (Figure 1(B)). Interestingly, bioinformatics analysis also demonstrated that death receptors, including DR4 and DR5, were overexpressed in CRC tumor tissues (Figure 1(A)), suggesting the potential of TRAIL as an anticancer agent for CRC. In fact, TRAIL showed robustly (ICs50 < 10 nM) cytotoxicity in CRC cells, including COLO205, HCT116, and LS174T cells overexpressing DR5 and/or DR4 (Figure 1(C, D)), indicating that TRAIL might overcome the chemotherapeutic MDR of CRC cells. Nevertheless, due to the low expression of death receptors, some CRC cells, such as HT29, showed moderate resistance (IC50s > 50 nM) to TRAIL (Figure 1(C, D)), suggesting the need to improve the cytotoxicity of TRAIL in these cells.

### Fusion to tumor-homing RGR peptide enhances the cell-binding and cytotoxicity of TRAIL in CRC cells

**Figure 2.**
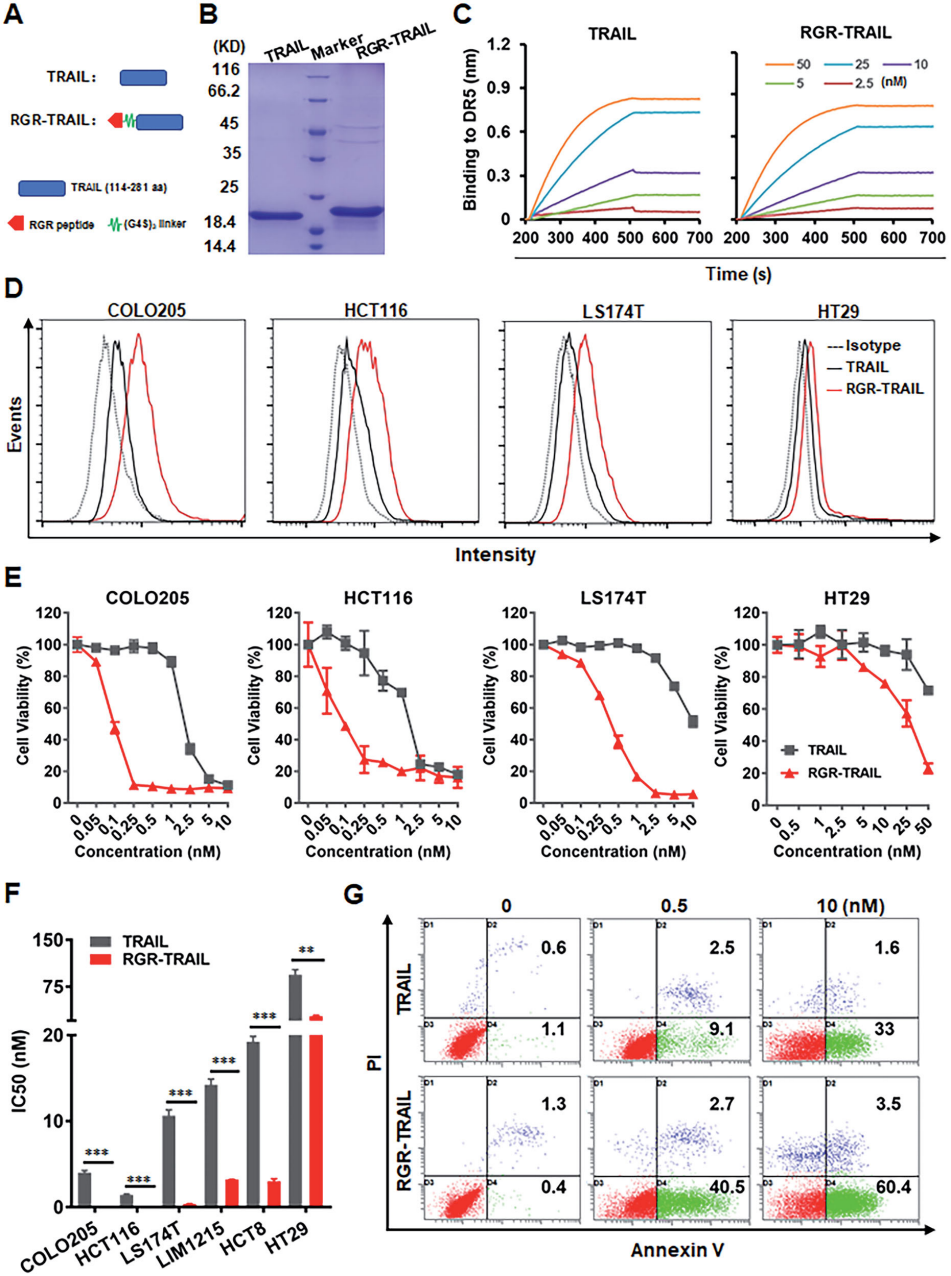
Fusion with the tumor-homing RGR peptide enhanced the cytotoxicity of TRAIL in CRC cells. (A) Schematic diagram of RGR-TRAIL. The tumor-homing RGR peptide was fused to the N-terminus of TRAIL to produce RGR-TRAIL. (B) SDS–PAGE of purified proteins. (C) Affinities for DR5 were measured by biolayer interferometry. DR5-Fc proteins were loaded on protein A-coated probes followed by insertion into a solution containing different concentrations (2.5–50 nM) of TRAIL proteins for the association. (D) Binding to CRC cells measured by flow cytometry. (E, F) Dose-dependent cytotoxicity (E) and IC50s (F) in representative CRC cells. (G) Apoptosis of COLO205 cells indicated by annexin V/PI dual staining combined with flow cytometry.

Tumor-homing modification might improve the cytotoxicity of TRAIL. To endow TRAIL with tumor-homing ability, the tumor-homing RGR peptide was introduced into the N-terminus of TRAIL to produce RGR-TRAIL (Figure 2(A)). SDS–PAGE showed that the molecular weight of RGR-TRAIL recovered from *E. coli* was approximately 22 KD, as compared to 20 KD of that of TRAIL (Figure 2(B)). Biolayer interferometry analysis showed that the affinities of RGR-TRAIL for DR4-Fc (1.1 nM) and DR5-Fc (0.46 nM) were comparable to those of TRAIL for DR4-Fc (1.5 nM) and DR5-Fc (0.89 nM) (Figure 2(C) and Supplementary Figure S2). The cytotoxicity of RGR-TRAIL and TRAIL was neutralized by exogenous death receptor proteins added into the cytotoxicity assay system to a similar degree (Supplementary Figure S3). These results indicated that fusion to the RGR peptide did not interfere with the binding of TRAIL to its death receptors.

Cell-binding assays showed that RGR-TRAIL bound more than TRAIL to CRC cells, including COLO205, HCT116, LS174T, and HT29 cells (Figure 2(D)). Accordingly, the cytotoxicity of RGR-TRAIL in the tested CRC cells was significantly greater than that of TRAIL. In particular, the IC50s of RGR-TRAIL for CRC cells, including COLO205, HCT116, LS174T, LIM1215, and HCT8, were 10–40 times lower than those of TRAIL for these cells (Figure 2(E, F)), indicating that fusion to the tumor-homing RGR peptide enhanced the cytotoxicity of TRAIL in CRC cells by improving its cell binding. Further analysis showed that both RGR-TRAIL and TRAIL-induced apoptosis in COLO 205 cells (Figure 2(G)). Caspase 3, 8, and 9 activities were detectable in COLO205 cells after treatment with RGR-TRAIL and TRAIL (Supplementary Figure S4(A)). Accordingly, the cytotoxicity of both RGR-TRAIL and TRAIL was reduced by the addition of a pan-caspase inhibitor or specific inhibitors of caspase 3, 8, and 9 into the cytotoxicity assay system (Supplementary Figure S4(B)). These results indicated that fusion with the tumor-homing RGR peptide improved the cytotoxicity but did not change the caspase-dependent apoptosis induction of TRAIL in CRC cells. Nevertheless, although the IC50s of RGR-TRAIL in HT29 cells were also lower (93.8 nM vs. 27.3 nM) than those of TRAIL, the cytotoxicity of RGR-TRAIL in HT29 cells was far from satisfactory, suggesting the need to further sensitize HT29 cells to TRAIL (Figure 2(F)).

### Fusion to tumor-homing RGR peptide improves the tumor uptake and antitumor effects of TRAIL in mice bearing CRC tumor xenografts

**Figure 3.**
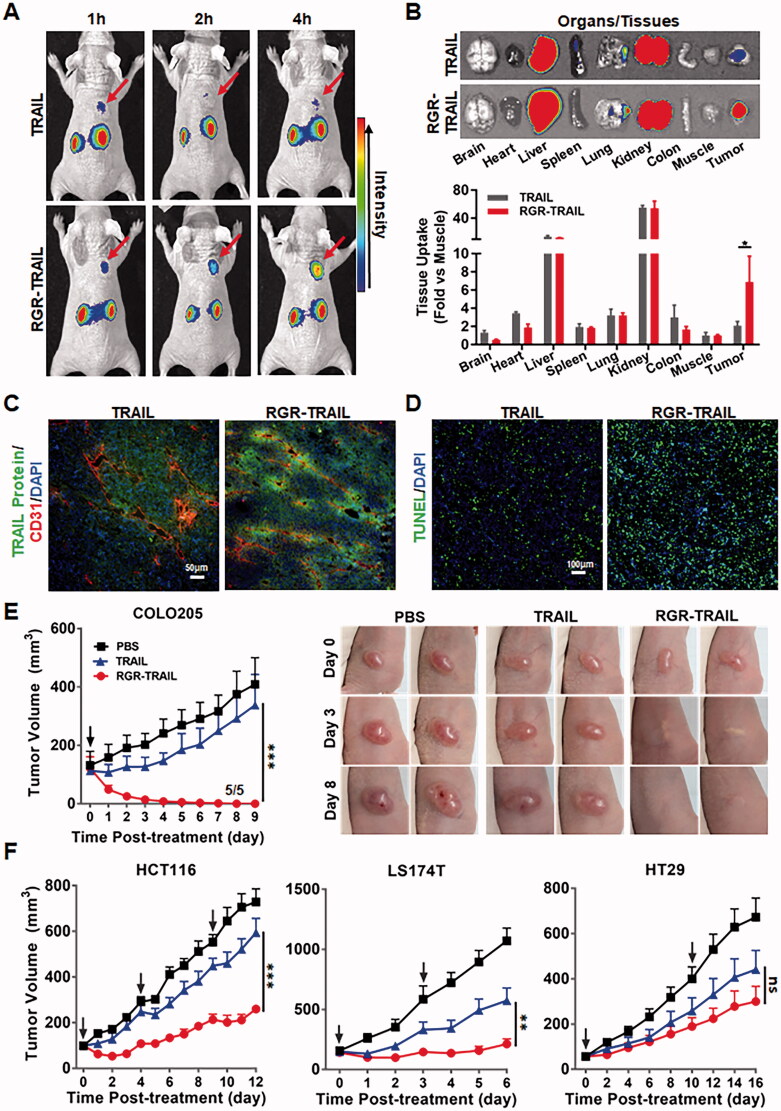
Fusion with the tumor-homing RGR peptide improved the tumor uptake and antitumor effect of TRAIL in mice bearing CRC tumor xenografts. (A) Tumor uptake of TRAIL proteins indicated by live optical imaging. CF750-labeled RGR-TRAIL and TRAIL (5 mg/kg) was intravenously injected into mice (*N* = 3) bearing COLO205 tumor xenografts followed by scanning the mice at different times (1–4 h) postinjection. (B) Biodistribution of RGR-TRAIL and TRAIL in mice bearing COLO205 tumor xenografts. The mice (*N* = 3) were sacrificed at 4 h postinjection of CF750-labeled TRAIL proteins. The tumor xenografts and some organs/tissues were collected and scanned simultaneously. (C) Cellular distribution of RGR-TRAIL. FAM-labeled RGR-TRAIL and TRAIL (10 mg/kg) were intravenously injected into mice bearing COLO205 tumor xenografts, followed by a collection of the tumor xenografts at 1 h postinjection for frozen sectioning. Blood vessels in tumor tissues were indicated by the anti-CD31 antibody. The nuclei of cells were visualized using DAPI. (D) Apoptotic cells in COLO205 tumor xenografts. RGR-TRAIL or TRAIL (5 mg/kg) was intravenously injected into mice bearing COLO205 tumor xenografts. After treatment overnight, tumor xenografts were collected and sectioned under frozen conditions followed by TUNEL staining. The nuclei of cells were visualized by DAPI staining. (E, F) Tumor growth suppression in mice (*N* ≥ 5) bearing COLO205 (E), HCT116, LS174T or HT29 (F) tumor xenografts. RGR-TRAIL or TRAIL (5 mg/kg, single dose for COLO205, three doses for HCT116, and two doses for LS174T and HT29) was intravenously injected into mice bearing tumor xenografts followed by measuring the tumor volume every day. The number of tumor-free mice is indicated in mice bearing COLO205 tumor xenografts.

To monitor the tumor uptake of TRAIL proteins, the mice bearing COLO205 tumor xenografts were dynamically scanned using an optical imaging system after injection of CF750-labeled proteins. As shown in Figure 3(A), dynamic optical imaging demonstrated that tumor uptake of RGR-TRAIL increased over time and peaked at 4 h postinjection. However, poor TRAIL was detected in tumor grafts within 4 h postinjection. In fact, biodistribution analysis at 4 h postinjection demonstrated that tumor uptake of RGR-TRAIL was 3 times greater than that of TRAIL, whereas the accumulation of RGR-TRAIL in other normal organs/tissues was similar to that of TRAIL (Figure 3(B)), indicating that fusion to RGR peptide significantly increased the tumor-homing ability of TRAIL. Further cellular distribution assays also verified that RGR-TRAIL accumulated more than TRAIL in tumor cells (Figure 3(C)). Accordingly, RGR-TRAIL induced greater apoptosis of tumor cells than TRAIL (Figure 3(D) and Supplementary Figure S5). These results demonstrated that fusion to the tumor-homing RGR peptide significantly increased the tumor uptake of TRAIL.

The *in vivo* antitumor effects of RGR-TRAIL and TRAIL were further evaluated in mice bearing tumor xenografts of either COLO205, LS174T, HCT116 or HT29 CRC cells. In mice bearing COLO205 tumor xenografts, after injection of a single dose of RGR-TRAIL, the tumor volume decreased from the second day, and all tumor grafts in 5 mice were eradicated within 10 days postinjection. However, injection of the same amount of TRAIL only showed slight tumor growth suppression (Figure 3(E)), indicating that the antitumor effect of RGR-TRAIL was significantly (*P* < .001) greater than that of TRAIL in mice bearing COLO205 tumor xenografts. Similarly, the tumor growth suppression mediated by RGR-TRAIL was also significantly (*P* < .001) greater than that mediated by TRAIL in mice bearing either LS174T or HCT116 tumor xenografts. Nevertheless, RGR-TRAIL showed greater, although not significant, tumor growth suppression than TRAIL in mice bearing HT29 tumor xenografts (Figure 3(F)). These results demonstrated that fusion with the tumor-homing RGR peptide significantly enhanced the antitumor effects of TRAIL in mice bearing TRAIL-sensitive CRC tumor grafts. However, fusion to the tumor-homing RGR peptide was not sufficient to overcome the resistance of some CRC cells, such as HT29, to TRAIL.

### EGFR-targeted PDT sensitizes CRC cells to RGR-TRAIL by upregulating death receptors

**Figure 4.**
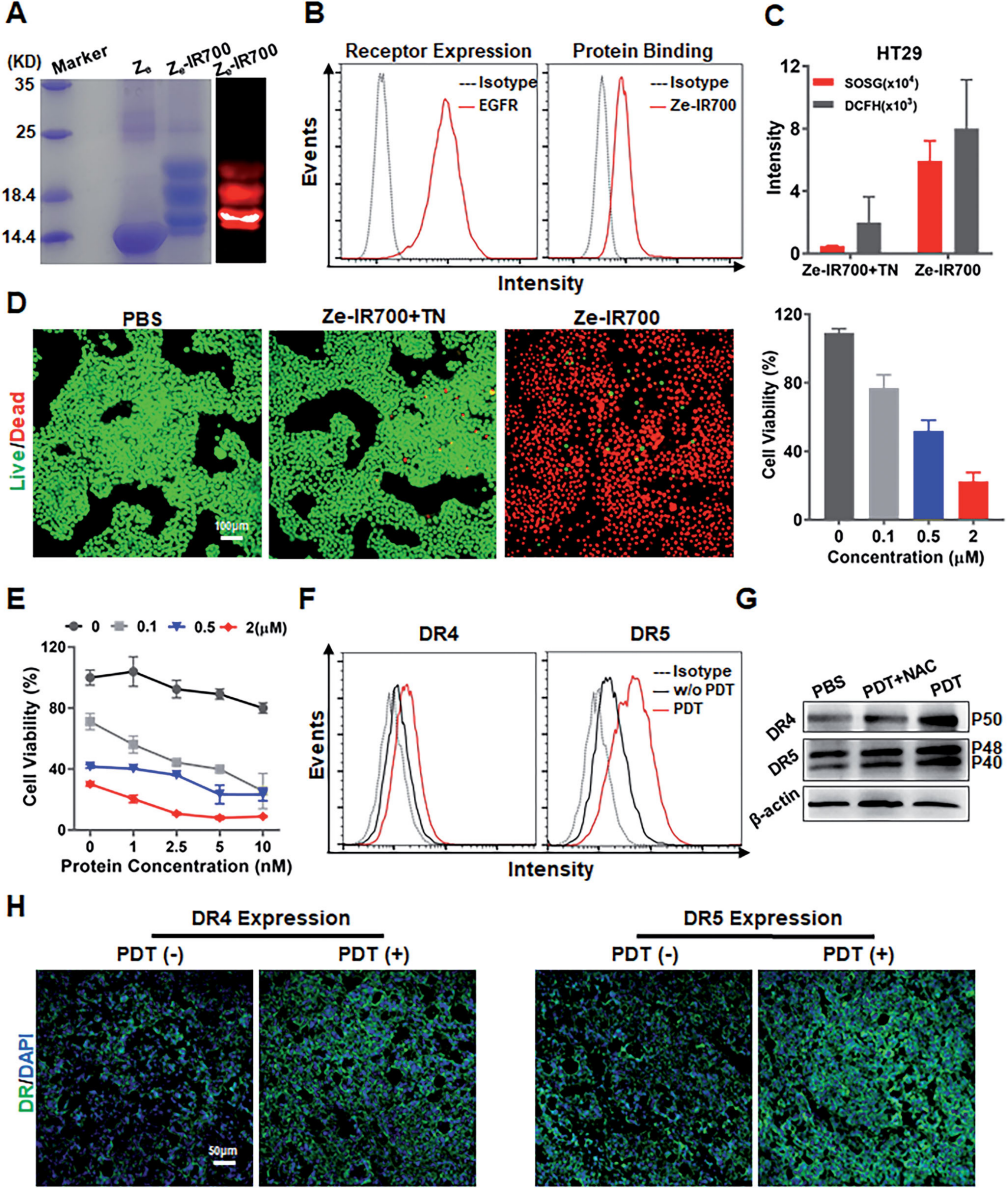
EGFR-targeted PDT sensitized CRC cells to RGR-TRAIL by upregulating death receptors. (A) Preparation of Ze-IR700 by conjugating photosensitizer IR700 to EGFR-specific Ze affibody. (B) Binding of Ze-IR700 to EGFR-expressing HT29 cells. (C) ROS produced in HT29 cells after treatment with PDT mediated by Ze-IR700 or Ze-IR700 digested with trypsin (Ze-IR700 + TN). (D) Phototoxicity of Ze-IR700-mediated PDT in HT29 cells. For PDT, cells were incubated with Ze-IR700 (1 μM) followed by washes with PBS prior to irradiation. Subsequently, live and dead cells were indicated as green and red under a fluorescence microscope, respectively, by dual staining with SYTO 9 and PI. Ze-IR700 digested with trypsin (Ze-IR700 + TN) was used as a control. To measure the dose-dependent phototoxicity, cells were incubated with increasing concentrations (0–2 μM) of Ze-IR700. After irradiation overnight, the number of surviving cells was measured by the CCK8 assay. (E) Cytotoxicity of RGR-TRAIL in HT29 cells pretreated with or without Ze-IR700-mediated PDT. Cells were incubated with increasing concentrations (0–2 μM) of Ze-IR700 prior to irradiation. Subsequently, increasing concentrations (0–10 nM) of RGR-TRAIL were added to the cells. (F) Expression of DR4 and DR5 in HT29 cells treated with or without Ze-IR700-mediated PDT. (G) Involvement of ROS in DR4 and DR5 expression after treatment with Ze-IR700-mediated PDT. To neutralize the ROS produced by PDT, NAC (4 mM) was added to the cells prior to irradiation. DR4 and DR5 in these cells were determined by western blotting. (H) Expression of DR4 and DR5 in HT29 tumor xenografts after treatment with (PDT(+)) or without (PDT(−)) Ze-IR700-mediated PDT for 2 h.

PDT might sensitize CRC cells to TRAIL. To perform tumor cell-targeted PDT, EGFR ubiquitously expressed in CRC cells was chosen as a target for tumor cell-targeted delivery of PS. As shown in Supplementary Figure S6(A), the EGFR-specific affibody Ze could bind cultured HT29 cells. Moreover, tissue (Supplementary Figure S6(B)) and cellular (Supplementary Figure S6(C)) distribution analyses demonstrated that the Ze affibody accumulated in HT29 tumor xenografts through binding to EGFR-expressing tumor cells, indicating that the Ze affibody could be used as a carrier for tumor cell-targeted delivery of PS. To perform tumor cell-targeted PDT, IR700 was conjugated to the Ze affibody to produce Ze-IR700 (Figure 4(A)). Flow cytometry analysis demonstrated that Ze-IR700 could bind EGFR-expressing HT29 cells (Figure 4(B)). To perform *in vitro* PDT, Ze-IR700 was incubated with HT29 cells followed by washes with PBS. Simultaneously, Ze-IR700 digested with trypsin (Ze-IR700 + TN) was used as a control. As shown in Figure 4(C), after irradiation, Ze-IR700-mediated PDT produced more ROS (as indicated by SOSG and DCFH) than trypsinized Ze-IR700-mediated PDT in HT29 cells. Accordingly, Ze-IR700, but not trypsinized Ze-IR700-mediated PDT, effectively killed HT29 cells (Figure 4(D)), indicating that the PDT-mediated damage of HT29 cells was Ze dependent.

To determine whether PDT sensitized HT29 cells, the viabilities of cells treated with monotherapy or combination therapy of Ze-IR700-mediated PDT and RGR-TRAIL were measured and compared. For combination therapy, cells were treated with PDT prior to the addition of RGR-TRAIL. As shown in Figure 4(E), compared with the viability of HT29 cells treated with monotherapy of either PDT (Ze-IR700) or RGR-TRAIL, the viability of HT29 cells treated with the combination therapy (Ze-IR700 + RGR-TRAIL) was definitely reduced. Accordingly, more cleaved caspase 3, 8, 9 and poly (ADP-ribose) polymerase (PARP) were detected in HT29 cells treated with combination therapy (Supplementary Figure S7). These results indicated that Ze-IR700-mediated PDT synergized with RGR-TRAIL in inducing apoptosis of HT29 cells. Flow cytometry analysis demonstrated that the positive rates of both death receptors of HT29 cells were increased (from 22.3% to 50.4% for DR4, from 7.5% to 21.7% for DR5) after PDT (Figure 4(F)). Western blotting verified that the PDT-induced increase in both DR4 and DR5 expression in cultured HT29 cells was ROS-dependent (Figure 4(H)). In addition, immunofluorescence also demonstrated the increased expression of both DR4 and DR5 in HT29 tumor xenografts after PDT (Figure 4(H)). These results indicated that Ze-IR700-mediated PDT could sensitize HT29 cells to RGR-TRAIL by upregulating death receptors.

### EGFR-targeted PDT enhances the antitumor effects of RGR-TRAIL in mice bearing CRC tumor xenografts

**Figure 5.**
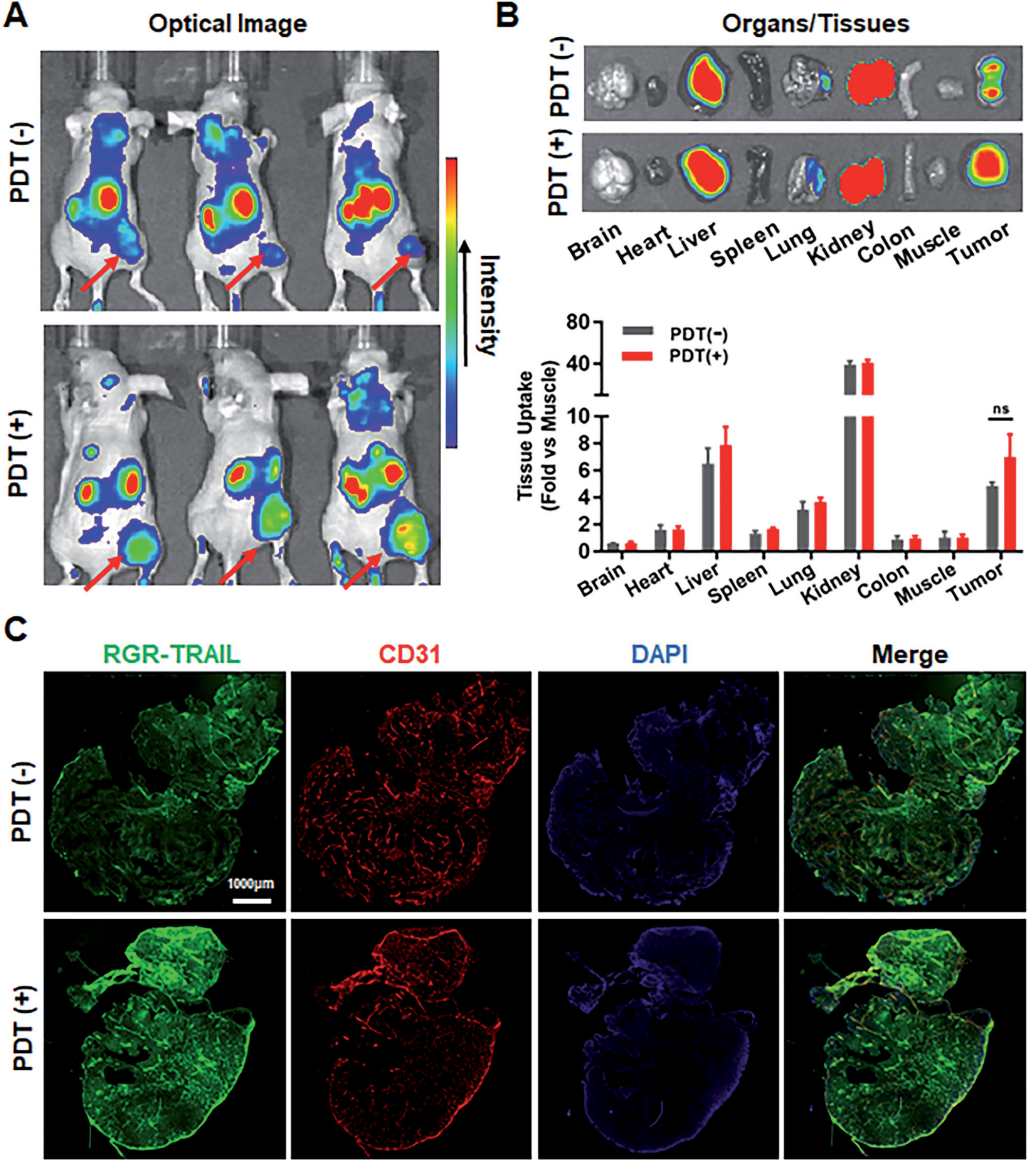
EGFR-targeted PDT increased the tumor uptake of RGR-TRAIL in mice bearing HT29 tumor xenografts. (A, B) Tumor uptake and biodistribution of RGR-TRAIL. CF750-labeled RGR-TRAIL (5 mg/kg) was intravenously injected into mice bearing HT29 tumor xenografts treated with (PDT(+)) or without (PDT(−)) Ze-IR700-mediated PDT followed by scanning the mice (A) or organs/tissues (B) with an optical imaging system at 4 h postinjection. (C) Cellular distribution of RGR-TRAIL in HT29 tumor xenografts. FAM-labeled RGR-TRAIL (10 mg/kg) was intravenously injected into mice bearing HT29 tumor xenografts treated with or without Ze-IR700-mediated PDT. Approximately 1 h later, the localization of RGR-TRAIL in these tumor xenografts was illustrated by visualizing cell nuclei with DAPI and blood vessels with an antibody against CD31.

**Figure 6.**
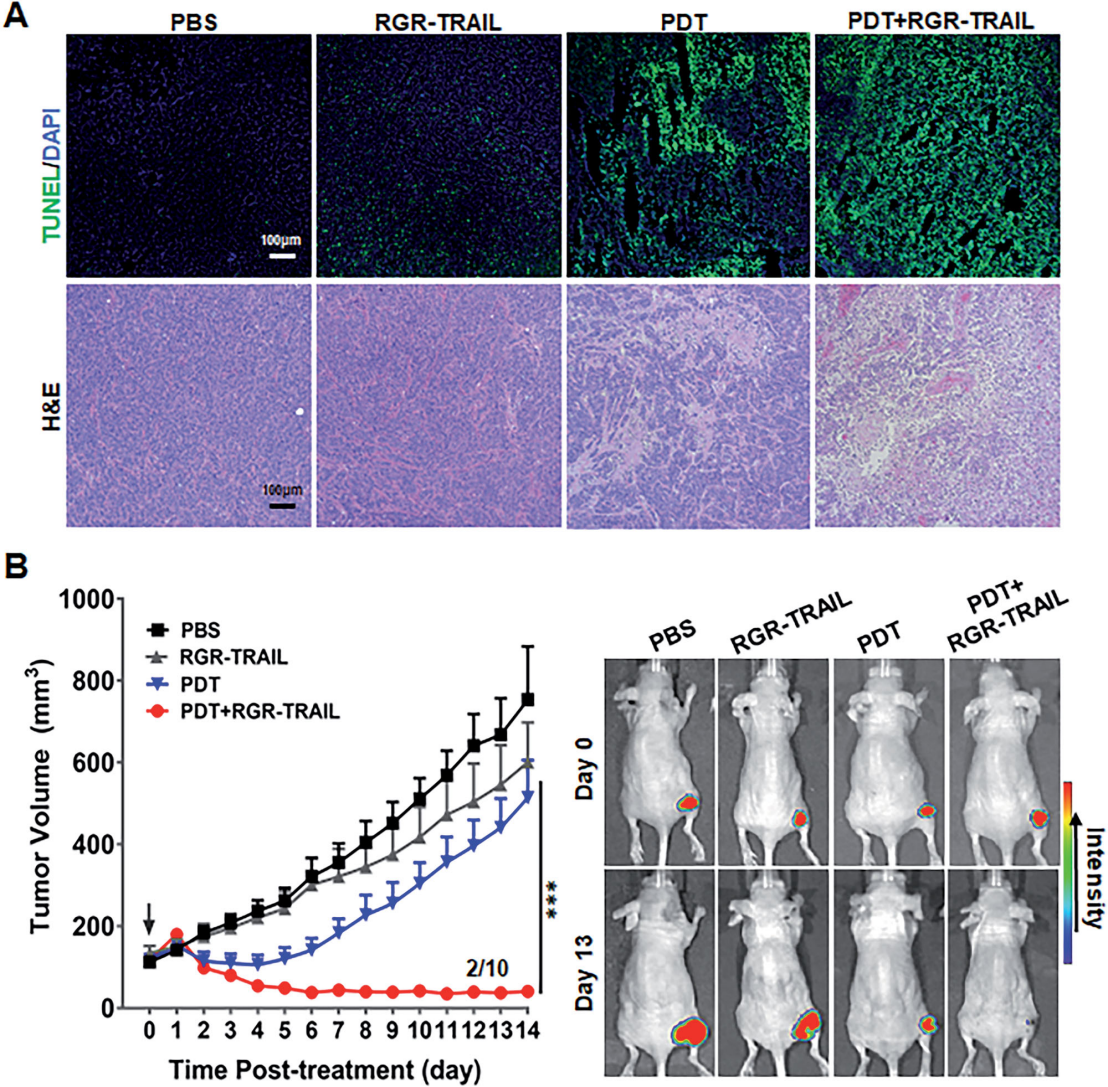
Combination therapy of RGR-TRAIL and EGFR-targeted PDT in mice bearing HT29 tumor xenografts. (A) Apoptosis induction and tissue damage mediated by monotherapy or combination therapy of RGR-TRAIL (5 mg/kg) and Ze-IR700-mediated PDT (1 mg/kg Ze-IR700, 60 J/cm^2^). After treatment overnight, apoptotic cells in tumor tissues were indicated by TUNEL, and tissue damage was visualized by H&E staining. (B) Tumor growth suppression mediated by monotherapy or combination therapy of RGR-TRAIL and Ze-IR700-mediated PDT. After treatment, the volume of HT29 tumor xenografts in mice (*N* ≥ 6) was measured daily. (C) Optical imaging of HT29 tumor xenografts treated with monotherapy or combination therapy of RGR-TRAIL and Ze-IR700-mediated PDT. For optical imaging, RFP-transgenic HT29 cells were subcutaneously injected into mice. After treatment, the mice were scanned daily.

In addition to sensitizing HT29 cells, Ze-IR700-mediated PDT that damages the tumor vascular system might also increase the *in vivo* antitumor effect by improving the tumor uptake of RGR-TRAIL. In fact, both optical imaging (Figure 5(A, B)) and cellular distribution (Figure 5(C)) demonstrated that RGR-TRAIL accumulated in tumor grafts treated with PDT more than that accumulated in tumor grafts treated without PDT, indicating that PDT truly increased the tumor uptake of RGR-TRAIL. Nevertheless, the PDT-mediated increase in tumor uptake of RGR-TRAIL was not significant (Figure 5(B)). However, Ze-IR700-mediated PDT greatly synergized with RGR-TRAIL in apoptosis induction and tissue damage in mice bearing HT29 tumor grafts (Figure 6(A)), suggesting that the synergy between PDT and RGR-TRAIL might be predominantly attributed to PDT-mediated sensitization of HT29 cells.

*In vivo* antitumor effect, evaluation demonstrated that combination therapy of Ze-IR700-mediated PDT and RGR-TRAIL exerted the strongest tumor growth suppression. The tumor grafts shrank from the 3^rd^ day after treatment with combination therapy and persisted with a small size in the following two weeks. Moreover, tumor grafts in 2 out of 10 mice were eradicated 10 days after treatment. However, monotherapy with Ze-IR700-mediated PDT and RGR-TRAIL showed only mild tumor growth suppression in mice bearing HT29 tumor xenografts (Figure 6(B) and Supplementary Figure S8(A)). At the end of the observation, the average volumes of tumor xenografts treated with PBS or monotherapy of Ze-IR700-mediated PDT (PDT) and RGR-TRAIL were 754.1 mm^3^, 514.4 mm^3^, and 600.9 mm^3^, respectively, compared with the 40.9 mm^3^ of that of tumor xenografts treated with combination therapy (PDT + RGR-TRAIL). Further optical imaging of mice bearing RFP-transgenic HT29 tumor xenografts verified that the combination therapy was superior to monotherapy of Ze-IR700-mediated PDT and RGR-TRAIL (Figure 6(C) and Supplementary Figure S8(B)). These results indicated that Ze-IR700-mediated PDT synergized with RGR-TRAIL in combating CRC cells with both chemotherapeutic MDR and TRAIL resistance. Notably, combination therapy of Ze-IR700-mediated PDT and RGR-TRAIL only induced transient (2–4 d) and slight (<5%) body weight loss in the early period after treatment (Supplementary Figure S9(A)). At the end of the observation, combination therapy did not cause obvious damage to the liver and kidney (Supplementary Figure S9(B)), indicating that combination therapy of Ze-IR700-mediated PDT and RGR-TRAIL-induced transient toxicity could be recovered within 2 weeks.

## Discussion

The high mortality of CRC patients was predominantly attributed to chemotherapeutic MDR mediated by ABC transporters overexpressed in CRC cells (Micsik et al., 2015; Xue et al., 2019). In fact, we found that several ABC transporters belonging to the ABCB, ABCC, and ABCG subfamilies were expressed at higher levels in CRC tumor tissues than in normal tissues (Figure 1(A), Supplementary Figure S1). Cytotoxicity assays revealed that all four tested CRC cell lines were simultaneously resistant to cisplatin, vincristine, doxorubicin, and bortezomib (Figure 1(B)), suggesting that conventional chemical agent-based monotherapies, even combination therapies, might be ineffective in combating CRC with MDR. Nevertheless, owing to different mechanisms of small chemicals, anticancer proteins might overcome the chemotherapeutic MDR of CRC. In fact, we found that the antitumor protein, i.e. TRAIL, exerted promising cytotoxicity in the tested CRC cells at low nanomolar concentrations (Figure 1(D)). In addition, DRs, including DR4 and DR5 for TRAIL, were overexpressed in tumor tissues from CRC patients (Figure 1(A)), indicating the potential of TRAIL for clinical therapy of CRC. Moreover, due to the ubiquitous expression of decoy receptors, normal cells are resistant to TRAIL (LeBlanc & Ashkenazi, 2003), which prompted us to develop TRAIL as a novel anticancer agent to overcome the MDR of CRC.

However, it was found that systemically administered TRAIL was trapped by normal cells overexpressing decoy receptors, which resulted in the low tumor uptake and subsequent inefficacy of TRAIL (LeBlanc & Ashkenazi, 2003; Merino et al., 2006). To improve the tumor-homing ability of TRAIL, directional molecules, such as antibody fragments and small peptides recognizing tumor-associated antigens, were fused to TRAIL (Möller et al., 2014; Tao et al., 2017). Due to the ease of overexpression in *E. coli*, small tumor-homing peptides belonging to the RGD and NGR families have been extensively used for TRAIL modification (Gong et al., 2017; Huang et al., 2017). As the RGR peptide has been used for the tumor-targeted delivery of tumor necrosis factor α (Johansson et al., 2012), we attempted to improve the tumor-homing ability of TRAIL by fuzing RGR to the N-terminus of TRAIL (Figure 2(A)). Protein-protein interactions demonstrated that RGR-TRAIL and TRAIL showed a similar affinity for both DR4 and DR5 (Figure 2(C), Supplementary Figure S2, and S3). However, the binding of RGR-TRAIL to CRC cells was significantly greater than that of TRAIL (Figure 2(D)), indicating that the RGR-mediated increase in the cell binding of TRAIL was DR independent. Usually, an increase in cell binding enhances the cytotoxicity of TRAIL in TRAIL-sensitive cells. In fact, the IC50s of RGR-TRAIL for CRC cells were 10–40 times lower than those of TRAIL in these cells (Figure 2(E, F)). In addition to the tested CRC cells, RGR fusion also significantly enhanced the cytotoxicity of TRAIL in liver, lung, pancreatic, and breast cancer cells. However, RGR fusion did not alter the cytotoxicity of TRAIL in normal cells, and both RGR-TRAIL and TRAIL showed little cytotoxicity in HUVECs (Supplementary Figure 10). These results suggested that RGR-TRAIL has the potential for a wide range of cancer therapies. Nevertheless, little is known about the target molecules for RGR. Platelet-derived growth factor receptor β (PDGFRβ) overexpressed on pericytes was suggested as a target molecule for RGR in a pancreatic cancer mouse model (Joyce et al., 2003). In fact, after incubation with PDGFRβ, pulldown of RGR-TRAIL, but not TRAIL was achieved (Supplementary Figure S11), indicating that PDGFRβ was a target molecule of RGR peptide in RGR-TRAIL. However, the human CRC cells tested in this paper expressed no or low levels of PDGFRβ (Tao et al., 2017), suggesting that the extensive enhancement in the *in vitro* binding of TRAIL to these CRC cells (Figure 2(D)) was not predominantly attributed to RGR-mediated PDGFRβ binding. *In vivo*, RGR-mediated binding to PDGFRβ-expressing pericytes (Joyce et al., 2003) might contribute to the enhancement of tumor uptake of RGR-TRAIL (Figure 3(A)). The enhancement in *in vitro* binding of RGR-TRAIL to CRC cells expressing no or little PDGFRβ suggested other molecular targets for RGR that need great identification works in the future. Although not all molecular targets of RGR in tumors have been defined, improvement in tumor-homing ability and antitumor effect of TRAIL demonstrated that RGR fusion is an alternative way for targeted delivery of anticancer proteins.

As TRAIL induces apoptosis of tumor cells by triggering DRs, the sensitivity of tumor cells to TRAIL is usually proportional to the level of DRs expressed on these cells (Wong, 2011; Di et al., 2013; Razeghian et al., 2021). In fact, COLO205 cells expressed more DR4 and DR5 than HT29 cells (Figure 1(B)). Accordingly, *in vitro* cytotoxicity assays demonstrated that COLO205 cells were more sensitive than HT29 cells to TRAIL and RGR-TRAIL (Figure 1(D)), Figure 2(E)). In mice, all COLO205 tumor xenografts were eradicated by intravenous injection of a single dose of RGR-TRAIL (Figure 3(E)). However, injection of two doses of RGR-TRAIL only showed mild tumor growth suppression in mice bearing HT29 tumor xenografts (Figure 3(F)), suggesting that the level of DR in these CRC cells was crucial for the antitumor effect of RGR-TRAIL; thus, increasing the DR level might sensitize HT29 cells to RGR-TRAIL. In fact, it was found that numerous chemical drugs could sensitize CRC cells to TRAIL by upregulating DR expression (Stolfi et al., 2012). However, most chemical drugs are cytotoxic to both tumor and normal cells, which limits the *in vivo* combination therapy of these chemical drugs and TRAIL. In addition to cytotoxic chemical drugs, Abrahamse & Houreld (2019) demonstrated that DR4 in Caco-2 CRC cells was upregulated by *in vitro* PDT (Abrahamse & Houreld, 2019). In this work, it was found that tumor cell-targeted PDT could simultaneously upregulate DR4 and DR5 in HT29 cells, thus drastically sensitizing HT29 cells to tumor-homing RGR-TRAIL (Figure 4(E–G)), which was consistent with our previous work (She et al., 2021). Accordingly, combination with tumor cell-targeted PDT significantly enhanced the *in vivo* antitumor effect of RGR-TRAIL in mice bearing HT29 tumor xenografts. Monotherapy with RGR-TRAIL and EGFR-targeted PDT showed only mild to moderate tumor growth suppression, whereas two out of ten tumor xenografts were eradicated by the combination therapy (Figure 6(B)). According to the significantly enhanced antitumor effect in mice, it might be innovative to administer a combination therapy of TRAIL proteins and tumor cell-targeted PDT in clinical therapy for refractory CRC.

Compared with nonspecific cytotoxic chemical drugs, tumor cell-targeted PDT takes advantage of controlling tissue damage by using targeted delivery of PS and subsequent local irradiation. Due to the metabolism and leakage of PS, normal organs/tissues, especially the liver and kidney, might be sensitized to TRAIL. According to the change in body weight, combination therapy of RGR-TRAIL and EGFR-targeted PDT did not obviously reduce the body weight of mice during the whole period of treatment (Supplementary Figure S9(A)). No abnormal structures were observed in the liver and kidney of mice treated with either monotherapy or combination therapy (Supplementary Figure S9(A)). These results support the value of further evaluating the combination therapy of RGR-TRAIL and tumor cell-targeted PDT in clinical therapy of CRC, especially in cases with MDR and TRAIL resistance. However, as shown in Supplementary Figure 12, although the blood clearance of RGR-TRAIL was slightly slower than that of TRAIL, the half-life of RGR-TRAIL (47 ± 3.9 min) was not significantly longer than that of TRAIL (31 ± 2.6 min), suggesting that RGR-TRAIL might also be limited by its short serum half-life. Evidently, additional work on extending the serum half-life of RGR-TRAIL is needed in the future.

## Conclusions

Chemotherapeutic MDR is common in CRC cells, whereas TRAIL induces apoptosis of many CRC cells. Fusion to the tumor-homing RGR-peptide increased the cytotoxicity of TRAIL in CRC cells overexpressing death receptors by enhancing cell binding. However, RGR fusion showed little improvement in cytotoxicity of TRAIL in CRC cells expressing low death receptors. Tumor cell-targeted PDT extensively sensitized CRC cells to the tumor-homing TRAIL variant by increasing death receptor expression, which resulted in the eradication of tumor xenografts of CRC cells with both chemotherapeutic MDR and TRAIL resistance. These results indicated that combination therapy of the novel tumor-homing TRAIL variant and tumor-cell targeted PDT might be developed as a novel strategy to combat refractory CRC.

## Supplementary Material

Supplemental Material
