# Supplementary material for: A novel tumor-homing TRAIL variant eradicates tumor xenografts of refractory colorectal cancer cells in combination with tumor cell-targeted photodynamic therapy
Source: Drug Deliv. 2022 May 30;29(1):1698–711. doi: 10.1080/10717544.2022.2079766 (PMC9176698; doi:10.1080/10717544.2022.2079766)
Supplement: Supplemental Material [file IDRD_A_2079766_SM6240.docx]

**Supplementary materials**

**A novel tumor-homing TRAIL variant eradicates tumor xenografts of refractory colorectal cancer cells in combination with tumor cell-targeted photodynamic therapy**

Zhao Li^1^*, Tianshan She ^1^*, Hao Yang^1,2,3^, Tao Su^4^, Qiuxiao Shi^1^, Ze Tao^1,2,3^, Yanru Feng^1^, Fen Yang^1^, Jingqiu Cheng^1,2,3,4^, Xiaofeng Lu^1,2,3^#

1 NHC Key Lab of Transplant Engineering and Immunology, West China Hospital, Sichuan University, Chengdu 610041, China

2 Sichuan Provincial Engineering Laboratory of Pathology in Clinical Application, West China Hospital, Sichuan University, Chengdu 610041, China

3 Frontiers Science Center for Disease-related Molecular Network, West China Hospital, Sichuan University, Chengdu 610041, China

4 Laboratory of Clinical Proteomics and Metabolomics, Institutes for Systems Genetics, West China Hospital, Sichuan University, Chengdu 610041, China

*These authors contributed equally to this work.

#Corresponding author, Xiaofeng Lu, E-mail: [xiaofenglu@scu.edu.cn](mailto:xiaofenglu@scu.edu.cn)

**
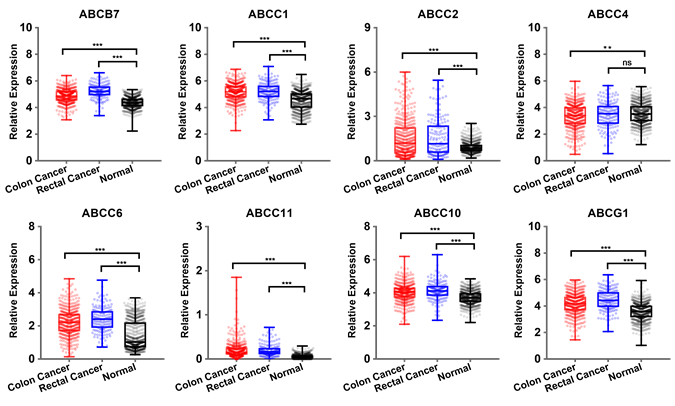
**

**Supplementary Figure S1** Expression of ABC transporters contributing to chemotherapeutic MDR and death receptor in tumor tissues derived from CRC patients.


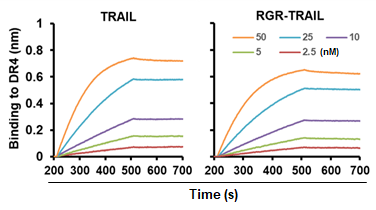


**Supplementary Figure S2** The affinity of RGR-TRAIL and TRAIL for DR4 was measured by biolayer interferometry. DR4-Fc proteins were loaded on protein A-coated probes followed by insertion into solution containing different concentrations (2.5-50 nM) of TRAIL proteins for association.


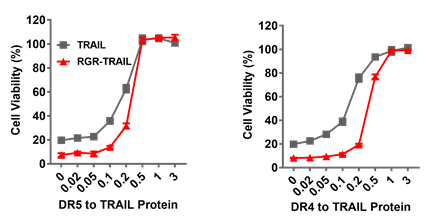


**Supplementary Figure S3** Neutralization of the cytotoxicity of RGR-TRAIL in COLO205 cells by exogenous death receptors. In the cytotoxicity assay system, increasing concentrations of DR4-Fc or DR5-Fc were mixed with constant RGR-TRAIL or TRAIL (20 nM) at different molar ratios (0-3) prior to addition into the cells.

**
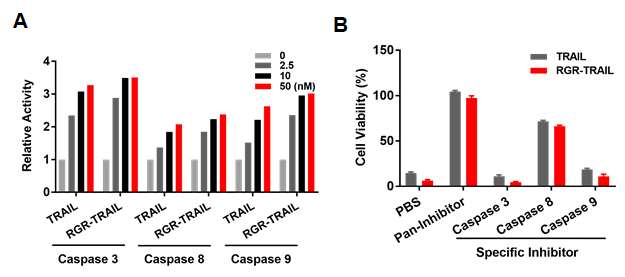
**

**Supplementary Figure S4** Involvement of caspase in RGR-TRAIL-induced apoptosis of COLO205 cells. (A) Activation of caspase 3, 8, and 9 in cells after treatment with different concentrations (0-50 nM) of RGR-TRAIL or TRAIL. The activity of activated caspase was measured using a colorimetric assay kit with specific substrates. (B) Inhibition of the cytotoxicity of RGR-TRAIL and TRAIL. Pancaspase inhibitor or specific inhibitor (20 μM) was added to the cells and preincubated for 2 h prior to the addition of TRAIL proteins.


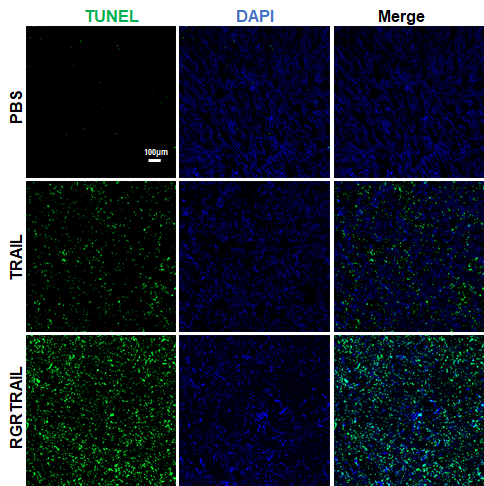


**Supplementary Figure S5** TRAIL protein-induced apoptosis in COLO 205 tumor xenografts. A single dose of 5 mg/kg RGR-TRAIL or TRAIL was intravenously injected into mice bearing COLO205 tumor xenografts. The apoptotic cells in tumor tissues were indicated by TUNEL at 25 h postinjection. The nuclei of cells were visualized by DAPI staining.


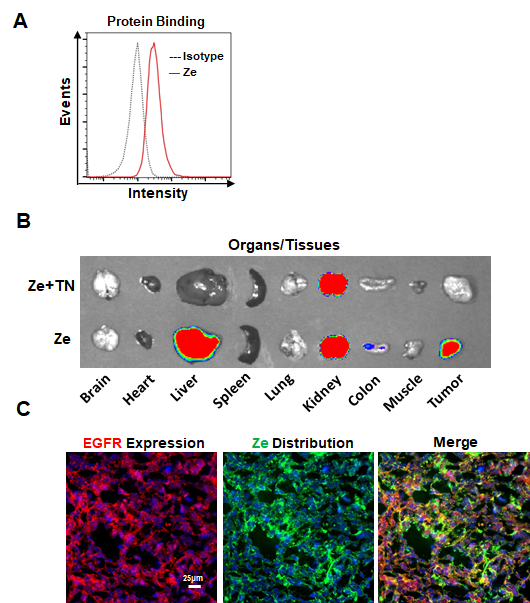


**Supplementary Figure S6** Tumor-homing characteristics of Ze affibody in mice bearing HT29 tumor xenografts. (A) Binding of the Ze affibody to HT29 cells. (B) Biodistribution of the Ze affibody in mice bearing HT29 tumor xenografts. CF750-labeled Ze affibody (Ze, 3 mg/kg) was intravenously injected into mice followed by scanning the tumor grafts and normal organs/tissues at 4 h postinjection. CF750-labeled Ze affibody digested with trypsin (Ze+TN) was used as a control. (C) Cellular distribution of the Ze affibody in HT29 tumor xenografts. FAM-labeled Ze affibody (10 mg/kg) was intravenously injected into mice bearing HT29 tumor xenografts. Subsequently, the tumor xenografts were collected at 4 h postinjection and sectioned under frozen conditions. Tumor cells were identified using an antibody against EGFR. DAPI staining was used to visualize cell nuclei.

**
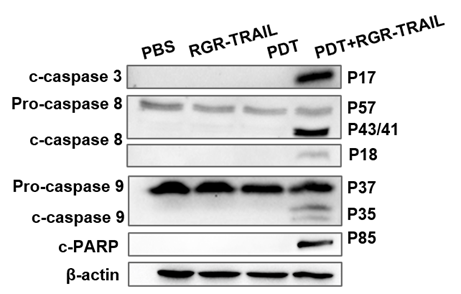
**

**Supplementary Figure S7** western blot of cleaved caspase 3 (c-caspase 3), cleaved caspase 8 (c-caspase 8), caspase 9 (pro-caspase 9, c-caspase 9) and cleaved PARP (c-PARP) in HT29 tumor cells treated with monotherapy or combination therapy of Ze-IR700-mediated PDT and RGR-TRAIL. β-actin was used as a loading control.


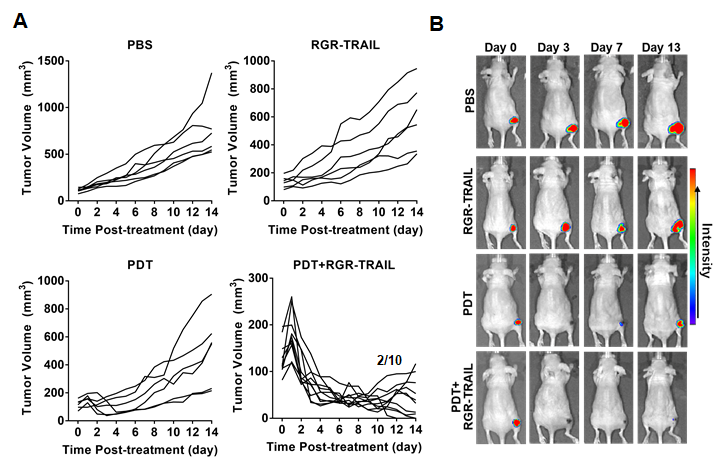


**Supplementary Figure S8** Combination therapy of RGR-TRAIL and EGFR-targeted PDT in mice bearing HT29 tumor xenografts. (A) Perspective tumor growth curve of the mice described in Figure 6B. (B) Optical images of mice bearing HT29 tumor xenografts described in Figure 6C.


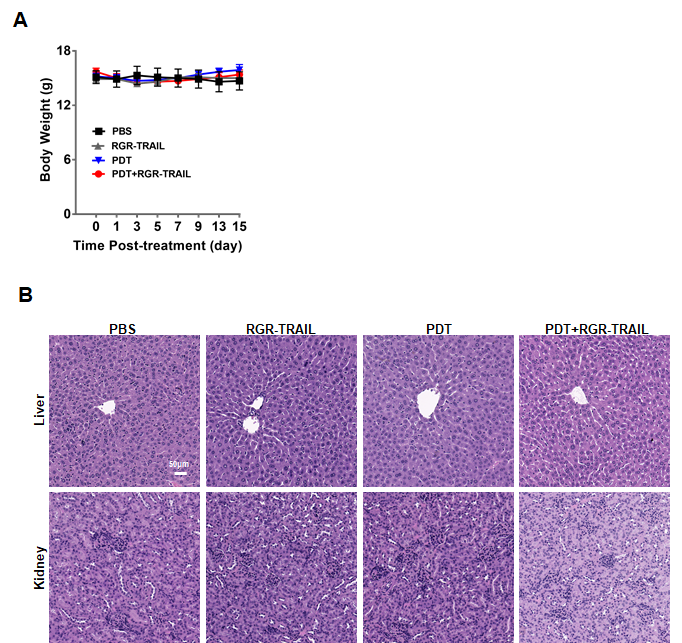


**Supplementary Figure S9** Acute toxicity of the combination therapy of Ze-IR700-mediated PDT and RGR-TRAIL in mice bearing HT29 tumor xenografts. (A) Body weights of mice after treatment. (B) Histochemistry of the liver and kidney from the mice sacrificed at the end of the observation.


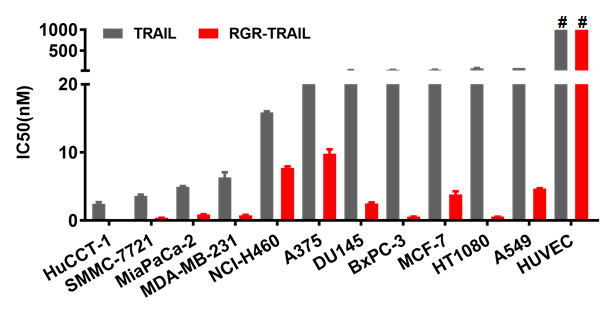


**Supplementary Figure S10** Cytotoxicity of RGR-TRAIL and TRAIL in non-CRC tumor cells and normal HUVECs. A total of 1-2×10^4^ cells in 100 μL medium were seeded in 96-well plates. After treatment with TRAIL proteins overnight, the number of surviving cells was measured by the CCK8 assay. The viability of cells treated with PBS was considered 100%. #IC50>1000nM.


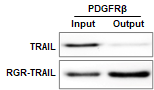


**Supplementary Figure S11** Binding of RGR-TRAIL and TRAIL to PDGFRβ. TRAIL proteins were pre-incubated with PDGFRβ-Fc (R&D, MN) at a molar ratio of 1:2 overnight at 4℃. Protein A/G agarose (GE healthcare, CA) was blocked with 5 mg/mL bovine serum albumin overnight. PDGFRβ-Fc proteins were adsorbed on protein A/G agarose and TRAIL proteins bound to PDGFRβ-Fc were visualized by western blot using an antibody against TRAIL.


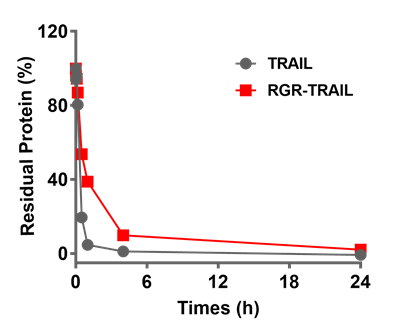


**Supplementary Figure S12** Pharmacokinetics of RGR-TRAIL and TRAIL in mice. The blood clearance of TRAIL proteins was measured by (ELISA). TRAIL proteins (10 mg/kg) were intravenously injected into the mice (N=3) followed by collection the blood samples at different time (0-24 h) postinjection. Residual TRAIL proteins in the plasma were measured by enzyme-linked immunosorbent assay with antibody against TRAIL. The relative residual protein was calculated as percentage of that in plasma collected immediately postinjection (0 min) that was considered as 100%.
